# School closures and teenage pregnancy

**DOI:** 10.2471/BLT.21.020121

**Published:** 2021-01-01

## Abstract

Pandemic-related school closures are impacting the sexual and reproductive health and rights of adolescent girls. Lynn Eaton reports.

It was the women’s shelter that brought it home to Najiba Khan, a sexual and reproductive health expert based in Kabul, Afghanistan. Khan (name changed at her request) travelled to the city of Herat in the west of the country in August 2020 to monitor the implementation of a programme designed to prevent and respond to violence against women.

What she found in the shelter were children. “Normally there should have been women there, but it was mostly little girls, some as young as nine,” she says. “Many of them were fleeing arranged marriages, and all had suffered some form of physical abuse.”

Khan visited the women’s shelter five months into Afghanistan’s national COVID-19 response. A key aspect of that response was the closure of all schools and educational institutions. In Khan’s judgement that decision contributed to the influx of girls at the shelter.

“Schools afford girls at least some degrees of protection from domestic abuse,” she says. “When the schools closed down, they had nowhere to go.”

School closures have been part of COVID-19 lockdown responses worldwide, with some 194 countries choosing to shut down schools and universities fully or partially since March 2020.

According to the United Nations Educational, Scientific and Cultural Organization (UNESCO) Institute for Statistics, at the height of the school closures in March of last year, an estimated 1.54 billion school and university students were sent home, representing 89% of the 1.73 billion young people enrolled in education globally. Some 743 million of those children were female.

“Nine months after their implementation, we are only beginning to measure the impact of school closures, but there are already indications of worrying trends,” says Veronica Kamanga Njikho, a Gender Programme Specialist at the United Nations Children’s Fund (UNICEF), also based in Kabul.

One concern is the possible contribution school closures make to unintended teenage pregnancy, the consequences of which are multiple and serious, including termination of education, reduced job and career prospects, and increased vulnerability to poverty and exclusion. Teenage pregnancy can also have negative impacts on health. Indeed, as pointed out by Anshu Banerjee, director of Maternal, Newborn, Child and Adolescent Health and Ageing at the World Health Organization (WHO), complications during pregnancy and childbirth are the leading cause of death for 15–19-year-old girls globally.

It will take time for the epidemiological picture regarding teenage pregnancies to become clear, and early reports of a significant pandemic-related spike in teenage pregnancies may be unreliable. For example, a widely reported 40% increase in teenage pregnancies in Kenya in the first three months of the country’s national lockdown appears not to be borne out by the available data.

“Schools are […] places of empowerment and learning, including learning about relationships, reproductive health, and the risk of pregnancy.”

Joannie Marlene Bewa

“The total number of teenage pregnancies across the country for the January to May period in 2020 was 151 433, which is significantly lower than the 175 488, reported for the same period in 2019,” points out Angela Nguku, executive director of the White Ribbon Alliance Kenya, a nongovernmental organization focused on reproductive, maternal, newborn and adolescent health and rights in the country.

Nguku notes, however, that it is possible that fewer teenage pregnancies were reported during the period in question because many young girls shied away from health facilities due to pandemic-related fears. She also points out that most adolescent mothers do not attend antenatal care, and that those who do tend to wait until the last trimester of their pregnancy. 

If school closures are having an impact on the rate of teenage pregnancies, it will not be the first time. Lisa Bos, Director of Government Relations at World Vision, a faith-based nongovernmental organization and member of UNESCO’s COVID-19 Global Education Coalition, cites the example of Sierra Leone during the Ebola virus disease outbreak of 2014–2015.

“Schools were closed for eight months during the outbreak and according to some estimates, teenage pregnancy rates doubled,” she says, acknowledging that the lack of reliable data makes it difficult to assess trends.

Like Khan, Bos believes schools provide an important safe haven for young girls. “Teachers generally keep an eye on the girls and can intervene if they identify signs of abuse,” she says. “When schools close, children are often left unsupervised and in the worst cases can be exposed to predatory family members and neighbours.”

A survey carried out by the White Ribbon Alliance in April and May 2020 appears to provide some anecdotal support for Bos’s assessment. Based on interviews with adolescent girls in Kenya, the study reported, for example, a marked increase in consensual sexual activity.

“The exercise revealed some stark realities regarding the sexual and reproductive health choices that adolescents are making during this COVID-19 time,” Nguku says. “Consensual sex appears to have seen a sharp increase with idleness and boredom cited as the main reasons for the increased activity.”

Bos believes that girls who are in school are also more likely to understand the consequences of sexual activity and have the confidence to stand up for themselves and fend off unwanted advances.

This is a view shared by public health expert, researcher and advocate for women’s health and rights, Joannie Marlene Bewa, who grew up in Benin and is now based in the United States of America. “Schools are a haven for many young girls,” she says, “but they are also places of empowerment and learning, including learning about relationships, reproductive health, and the risk of pregnancy.”

For girls who do become pregnant, the doors of schools are often slammed shut. This is what happened in Sierra Leone, where the government issued a countrywide ban on the re-enrolment of pregnant girls in April 2015, just before schools reopened after the Ebola outbreak.

Even in countries where readmission of pregnant girls is a legal requirement, not all of those girls go back to school. In Kenya, for example, legislation was passed in 1994 obliging schools to accept pregnant girls, but according to Nguku it is not always implemented. “Not all schools are aware of the 1994 law,” Nguku says, “but in many cases the girls themselves stay away from school because they are ashamed and made to feel ashamed by their families, communities and their teachers.”

“Lessons from the past need to be learned.”

Lisa Bos

In some instances, school closures put girls on the path to early marriage, which can also feed into domestic abuse and early pregnancies. However, as WHO’s Banerjee points out, increasing poverty – widely observed in the ongoing pandemic, as pandemic response measures limit economic activity – is probably a bigger driver. “In times of economic hardship, families are much more likely to get their daughters married to reduce financial hardship on their own family,” Banerjee says.

UNICEF’s Njikho concurs. “The link between poverty and early marriage is well established, and I fully expect to see an increase in early marriages in Afghanistan as part of the socioeconomic fallout of the pandemic,” she says.

For Bewa effective responses to these different issues will require cross-sectoral efforts. “Where schools continue to be closed (as of late November an estimated 952 million students were still being affected by school closures, according to UNESCO) we need to collaborate across the health and education sectors, making sure that girls can continue to be engaged in learning – virtually where access to digital technologies allows.”

Bewa argues for free phone lines for access to birth control and tests for sexually transmitted diseases and highlights the value of emerging social media initiatives. “A lot of countries have been using social media to get important messages about sexual and reproductive health out,” she says. “In Benin, for example, there is an app called *Ma Vie Mon Choix* (My Life My Choice) which educates young people about responsible sexuality.”

Bewa also emphasizes the need for reinforced material support in the form of contraceptives and feminine hygiene products and argues for ‘dignity kits’, which include sanitary products, to be made routinely available, rather than just as a part of humanitarian aid.

Clearly, there is work to be done on many fronts, but governments can have an immediate impact by making sure that the doors to schools remain open to adolescent mothers, including those who have become pregnant during school closures. “Governments should eliminate policies and practices that exclude pregnant girls from schools and facilitate the continued education of pregnant girls and adolescent mothers,” says World Vision’s Lisa Bos. “Lessons from the past need to be learned.”

**Figure:**
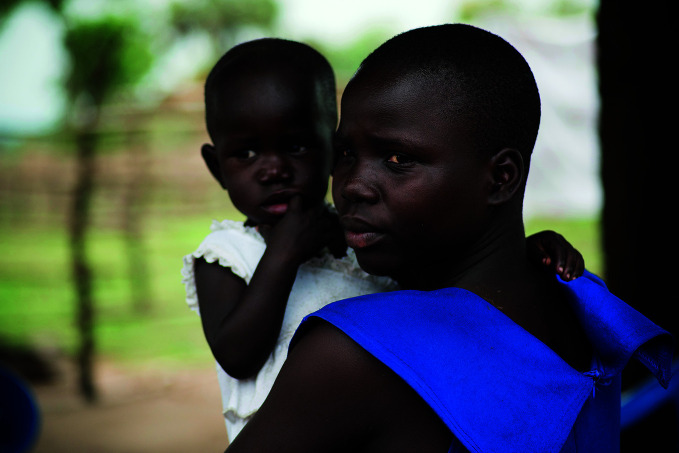
17-year-old beneficiary of a UNICEF programme supporting the return of girls to school after giving birth.

UNICEF/Henry Bongyereirwe

**Figure:**
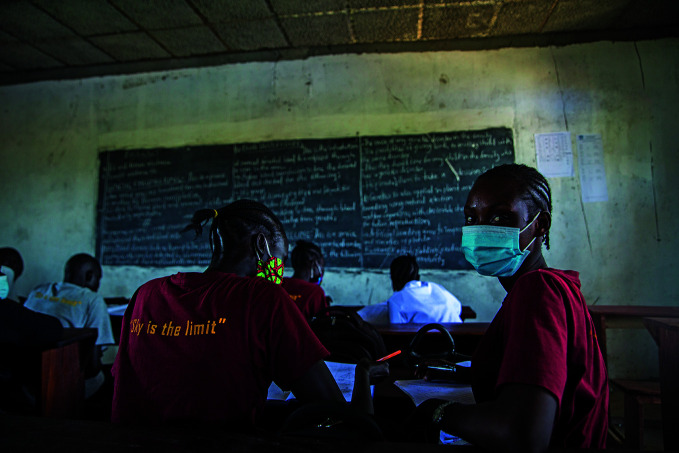
16-year-old girls return to school in Juba, South Sudan after a six-month school closure.

UNICEF/Bullen Chol

